# Prevalence and Characteristics of Emergency Department Visits by Pregnant People: An Analysis of a National Emergency Department Sample (2010–2020)

**DOI:** 10.5811/westjem.60461

**Published:** 2024-04-02

**Authors:** Carl Preiksaitis, Monica Saxena, Jiaqi Zhang, Andrea Henkel

**Affiliations:** * Stanford University School of Medicine, Department of Emergency Medicine, Stanford, California; † Stanford University School of Medicine, Department of Obstetrics and Gynecology, Stanford, California

## Abstract

**Introduction:**

The number and characteristics of pregnant patients presenting to the emergency department (ED) has not been well described. Our objective in this study was to determine the prevalence and characteristics of pregnant patients presenting to EDs in the US between 2010–2020.

**Methods:**

We completed a retrospective, cross-sectional study of patient encounters at hospital-based EDs in the US from 2010–2020. Using the ED subsample of the National Hospital Ambulatory Medical Care Survey (NHAMCS) we identified ED visits for female patients aged 15–44 years. We defined a subsample of these as visits for pregnant patients using discharge diagnosis codes specific to pregnancy. We compared this population of pregnant patient visits to those for non-pregnant patients and computed point estimates for nationally weighted values. Multivariable linear regression was used to determine factors independently associated with pregnant patient visits.

**Results:**

The 2010–2020 NHAMCS dataset included 255,963 ED visits. Of these visits 59,080 were for female patients 15–44 years old, and 6,068 of those visits were for pregnant patients. Pregnant patients accounted for 3% (95% confidence interval [CI] 2.7–3.2) of all ED visits and 8.6% (95% CI 8–9.3) of all visits among female patients 15–44 years. Weighting to a national sample, this equates to 2.77 million pregnant patients presenting for ED visits annually. Pregnant patients were more likely to be Black, Hispanic, or to use public insurance.

**Conclusion:**

Pregnant patients make up a significant number of ED visits annually and are more likely to be people of color or publicly insured. Interventions to address the effects of changing abortion legislation on emergency medicine practice may benefit from consideration that certain populations of pregnant people are more likely to present to the ED for care.

## INTRODUCTION

### Background

In June 2022, the US Supreme Court ruled on *Dobbs v Jackson Women’s Health Organization* and determined that there is no constitutional right to abortion, allowing individual states to legislate abortion restrictions.^1^ This decision has multiple anticipated implications for emergency clinicians, including an increase in pregnant patients presenting to the emergency department (ED) as a result of barriers to care, complications of self-managed abortions, or delayed presentation of emergent diagnosis due to fear of legal repercussions.^2^

### Importance

Use of the ED is high among pregnant patients, with studies showing that approximately 35% of these patients will visit the ED at least once during their pregnancy.^3^^,^^4^ These patients are more likely to be of racial and ethnic minorities, publicly insured, and have barriers to prenatal care access.^3^^,^^5^^,^^6^ Less is known, however, about the total population of pregnant patients who present to the ED. A secondary analysis of the 2006–2016 National Hospital Ambulatory Medical Care Survey (NHAMCS) identified that there were approximately 900,000 visits to the ED for early pregnancy loss, but the total population of pregnant patients was not described.^5^

Older cohort studies disagree on the pregnancy rate among reproductive-capable female patients, reporting values ranging from 2.3–33%.^7^^–^^9^ These studies report that many pregnancies are first identified in the ED, but the rate of incidental pregnancy in more recent years and how often these patients are provided with counseling has not been described.^10^ In fact, before the repeal of *Roe* *v Wade* there was an identified need for further emergency physician training in patient-centered reproductive healthcare.^5^^,^^10^ With increased legal restrictions, the need for emergency medicine policy and physician education has never been greater.^2^^,^^11^ To do this successfully, we must have a better understanding of the population that will be affected by these changes: pregnant people. This has not been recently reported in the literature, which led us to undertake this study.

### Goals of this Investigation

The primary objective of our study was to identify the prevalence of and characterize pregnant patients presenting to US EDs between 2010–2020.

## METHODS

### Study Design and Data Source

We completed a retrospective, cross-sectional study of patient encounters at hospital-based EDs in the US from 2010–2020. This period was selected as 2010 saw the passage of the Affordable Care Act, which had many effects for expanding healthcare and contraceptive coverage for women.^12^ Data was from the publicly available ED subsample of the NHAMCS.^13^ The NHAMCS is a survey conducted annually by the National Center for Health Statistics (NCHS), a part of the US Centers for Disease Control and Prevention (CDC).^14^

The NHAMCS uses a three-stage probability sampling design in an effort to provide a representative sample of all EDs in the country.^14^ First, 112 geographic probability sampling units, determined by various counties, towns, and cities are chosen. These are selected to be representative of different geographical regions and urban and rural areas. Within these sampling units, 450–500 short-stay hospitals (average length of stay fewer than 30 days) are sampled to ensure a diversity of hospital size and type. Finally, EDs that provide unscheduled care 24 hours a day/7 days a week at these sampled hospitals are selected for inclusion. Each included ED has visit data recorded over a randomly assigned 4-week period. During this period, data from selected visits is abstracted from the chart and entered into an electronic form by trained census takers.

Further description of the NHAMCS survey is available on the NCHS website.^14^ The NHAMCS produces a dataset that reflects a broad spectrum of ED visits. Using the survey’s sampling design, each data entry is assigned a weight to account for the relative contribution of that entry to the larger sample. As a result, each data point in the dataset represents a varying number of actual visits, depending on its assigned weight.

### Study Population

To determine our study population, we identified the total ED visits for NHAMCS between 2010–2020 for all patients. Visits were selected for women of reproductive age, defined by the CDC as being between ages 15–44 years.^15^ We acknowledge that research surrounding pregnancy often assumes cisgender identities, which may not describe people who are transgender or non-binary. We attempted to use language that is as inclusive; however, the data analyzed in this study uses gender labels that cannot be changed while remaining accurate to the source material.

### Definition of Pregnancy

We defined visits for pregnant patients within our cohort as those visits that had an International Classification of Diseases Revisions 9 or 10 (ICD-9 and -10) diagnosis code specific to pregnancy as one of the discharge diagnoses (eg, ectopic pregnancy; excessive vomiting in pregnancy; pregnant state, incidental). Specific diagnosis codes used for patient identification are listed in Appendix 1. These were initially filtered by SPSS Statistics v27 (IBM Corporation, Armonk, NY) and were then hand-verified by one study author (CP). We excluded encounters without ICD diagnosis codes or coded only as “elopement” or “left without being seen.”

### Pregnancy Identified in the Emergency Department

As this has important implications for emergency clinicians, a secondary goal of our analysis was to identify an estimate of the incidence of new pregnancy diagnosis in the ED—that is, visits where patients were first identified as pregnant during their ED visit. We defined a subset of pregnant patient visits as “incidental pregnancy” through ED reason-for-visit (RFV) codes and whether pregnancy was tested for in the ED. The RFV codes include the chief complaint, as well as other symptoms or medical problems related to the ED visit.^16^ We examined RFV codes and excluded patient visits with codes that suggested a previous diagnosis of pregnancy (eg, 1790.0 Problems and other conditions related to pregnancy; 2735.0 Diagnosed complications of pregnancy and puerperium). With the goal of obtaining a conservative estimate, we also excluded patient visits with RFV codes for vaginal bleeding. Patient visits from this group that had a pregnancy test sent in the ED made up our “incidental pregnancy” population. To obtain evidence of construct validity, we examined the ICD-9 and ICD-10 discharge codes for the “incidental pregnancy” population to ensure they were consistent with a new pregnancy diagnosis.

### Characteristics of ED Visits

Available demographics included patient age; race/ethnicity (non-Hispanic Black, non-Hispanic White, Hispanic, and other); payment/insurance status (private insurance, public insurance [Medicare, Medicaid or other state-based program], self-pay, and other or unknown insurance); and residence (private, unhoused, or other).

Visit characteristics included day of the week the visit took place (weekend or weekday), season (Fall, Winter, Spring, or Summer), and year of visit. Visit characteristics also included hospital admission, whether a pregnancy test was sent, use of ultrasound, consultation, length of visit, wait time to see a clinician, and return visit within 72 hours. Pregnancy test, ultrasound use, hospital admission, consultation, and return visit within 72 hours were dichotomous variables, and return visit referred to whether the patient was seen in the same ED in the prior 72 hours for any reason. We defined wait time as the time from arrival to first clinician contact, and length of visit was defined as the time from arrival to discharge. We analyzed both values as continuous variables.

Hospital-level characteristics included geographic region (Midwest, Northeast, South, and West) and metropolitan statistical area, reflecting an urban vs rural location as defined by the US Office of Management and Budget.

### Data Analysis

We followed the Strengthening the Reporting of Observational Studies in Epidemiology (STROBE) reporting guidelines.^17^ We descriptively analyzed ED visits for pregnant patients and determined the proportion of ED visits for pregnant patients among all ED visits (for women and men) as well as among women 15–44 years old. Demographic, visit, and hospital characteristics of presentations of pregnant patients among women of reproductive age were similarly analyzed. We compared the characteristics of visits for pregnant reproductive-aged women to those for non-pregnant reproductive-aged women using chi-squared tests for categorical factors and two-sample *t*-test for continuous variables. Statistical significance was set at *P* < 0.01 as recommended by NHAMCS documentation.^13^

We compared characteristics of visits for pregnant patients to non-pregnant patients using multivariable logistic regression. We examined unadjusted associations and then used multivariable logistic regression models to determine factors independently associated with visits for pregnant patients. Models generated odds ratios (OR) and 95% confidence intervals (CI). Statistical calculations were completed with SAS OnDemand for Academics (SAS, Inc, Cary, NC).

Weighted results representative of all ED visits rounded to the nearest thousand in the US were presented for analysis unless otherwise stated, as recommended by NHAMCS.^13^^,^^18^ Based on best practices for the use of NHAMCS data in research, we ensured that all reported estimates were based on >30 unweighted records, had a relative standard error of <30%, and did not include any items with a non-response rate >30% in our analysis.^14^^,^^18^ The NHAMCS imputes data for missing values in age, gender, race, and ethnicity using a model-based single, sequential regression method.^13^ Race and ethnicity had the highest average proportion of missing values in our dataset (17% and 21%, respectively); therefore, we performed a sensitivity analysis to ensure result durability.

### Ethics

Data from the NHAMCS are de-identified and publicly available. Use of this data for research purposes has been reviewed and approved by the National Center for Health Statistics Ethics Review Board (Protocol #2021-03).

## RESULTS

The 2010–2020 NHAMCS dataset included 255,963 visits (weighted n = 1,502,215,000, 95% CI 1,342,435,000–1,661,995,000), including 59,080 visits among women ages 15–44 (weighted n = 353,012,000, 95% CI 310,947,000–395,078,000). A total of 6,068 of these visits were for pregnant patients (weighted n = 30,489,000, 95% CI 26,117,000–34,861,000). Pregnant patient visits accounted for 3.0% (95% CI 2.7–3.2) of all ED visits. This equates to 2.77 million pregnant patients presenting for ED visits annually. Limiting the population to women ages 15–44 years, pregnant patient visits accounted for 8.6% (95% CI 8–9.3) of all ED visits.

Incidental pregnancy was identified in 672 patient visits (weighted n = 4,056,000, 95% CI 3,323,000–4,789,000). Incidental pregnancy visits accounted for 13.3% (95% CI 12.7–13.7) of all pregnant ED visits. Annually, this equates to 368,000 (95% CI 352,000–379,000) visits where pregnancy is diagnosed in the ED. The majority (52%) of the ICD-9 and −10 codes for these visits were for pregnancy-related complaints (eg, hyperemesis gravidarum, infection of the genital tract in pregnancy), and the remainder were diagnoses of pregnancy (eg, encounter for supervision of normal first pregnancy, pregnant state, incidental), aligning with our assumption that these visits represented new pregnancy diagnoses.

### Study Population Characteristics

The median age of women presenting with pregnancy was 25 years (interquartile range 21–30 years), with over half (57.9%) between the ages of 20–29 years (Table 1). Visits for pregnant patients were more likely to be for Black (31.6% vs 26.3% for non-pregnant women) and Hispanic (22.0% vs 15.5%) women. Pregnant patient visits were more likely to use public insurance than non-pregnant visits.

**Table 1.** Characteristics of female patients aged 15–44 years old presenting to the emergency department for care, 2010–2020, weighted and stratified by pregnancy status.

	Pregnant (n = 30,489,000)	Non-pregnant (n = 322,524,000)	*P*-value
*Patient characteristics*			
Age	25 (21–30)	28 (22–36)	<0.001
Age			<0.001
15–19 years	4,185,000 (13.7)	47,282,000 (14.7)	
20–29 years	17,640,000 (57.9)	124,098,000 (38.5)	
30–39 years	7,802,000 (25.6)	103,558,000 (32.1)	
40–44 years	862,000 (2.8)	47,586,000 (14.8)	
Race/ethnicity			<0.001
Non-Hispanic White	13,199,000 (43.3)	178,581,000 (55.4)	
Non-Hispanic Black	9,642,000 (31.6)	84,808,000 (26.3)	
Hispanic	6,695,000 (22.0)	49,863,000 (15.5)	
Non-Hispanic Other	952,000 (3.1)	9,271,000 (2.9)	
Payment source			<0.001
Private insurance	8,039,000 (26.4)	96,150,000 (29.8)	
Public insurance	15,197,000 (49.8)	133,820,000 (41.5)	
Self-pay	3,289,000 (10.8)	46,446,000 (14.4)	
Other	1,164,000 (3.8)	13,203,000 (4.1)	
Unknown	2,800,000 (9.2)	32,905,000 (10.2)	
Residence			0.21
Private residence	29,464,000 (96.6)	309,823,000 (96.1)	
Homeless	76,000 (0.2)	1,556,000 (0.5)	
Other	226,000 (0.7)	3,517,000 (1.1)	
Unknown	723,000 (2.4)	7,628,000 (2.4)	
*Visit characteristics*			
ED visit day			0.86
Weekend	8,087,000 (26.5)	86,105,000 (26.7)	
Weekday	22,403,000 (73.5)	236,419,000 (73.3)	
ED visit season			0.44
Fall	7,503,000 (24.6)	85,173,000 (26.4)	
Winter	7,735,000 (25.4)	77,058,000 (23.9)	
Spring	7,536,000 (24.7)	81,156,000 (25.2)	
Summer	7,715,000 (25.3)	79,136,000 (24.5)	
Year			<0.001
2010	2,453,000 (8.0)	17,134,000 (5.3)	
2011	2,716,000 (8.9)	32,089,000 (9.9)	
2012	2,363,000 (7.8)	30,316,000 (9.4)	
2013	2,362,000 (7.7)	30,350,000 (9.4)	
2014	2,570,000 (8.4)	32,656,000 (10.1)	
2015	3,028,000 (9.9)	31,333,000 (9.7)	
2016	3,073,000 (10.1)	31,538,000 (9.8)	
2017	2,902,000 (9.5)	30,809,000 (9.6)	
2018	2,710,000 (8.9)	27,058,000 (8.4)	
2019	3,558,000 (11.7)	31,182,000 (9.7)	
2020	2,743,000 (9.0)	28,059,000 (8.7)	
Hospital admittance	2,147,000 (7.0)	15,048,000 (4.7)	<0.001
Pregnancy test	13,665,000 (44.8)	87,455,000 (27.1)	<0.001
Ultrasound	13,423,000 (44.0)	18,889,000 (5.9)	<0.001
72-hour revisit	1,293,000 (4.2)	12,776,000 (4.0)	0.03
Seen by consultant	3,498,000 (11.5)	19,499,000 (6.0)	<0.001
Length of visit			<0.001
<1 hr	1,769,000 (7.6)	29,062,000 (11.8)	
1–2 hr	3,221,000 (13.9)	64,011,000 (26)	
2–4 hr	10,413,000 (44.9)	93,180,000 (37.8)	
>4 hr	7,789,000 (33.6)	60,415,0000 (24.5)	
Wait time			0.20
<30 min	15,538,000 (59.5)	172,983,000 (61.6)	
30 min-1 hr	4,830,000 (18.5)	50,720,000 (18.1)	
1–2 hr	3,475,000 (13.3)	35,543,000 (12.7)	
>2 hr	2,280,000 (8.7)	21,565,000 (7.7)	
*Hospital characteristics*			
Geographic region			0.38
Northeast	4,241,000 (13.9)	51,007,000 (15.8)	
Midwest	7,302,000 (23.9)	73,129,000 (22.7)	
South	12,631,000 (41.4)	130,591,000 (40.5)	
West	6,315,000 (20.7)	67,797,000 (21.0)	
Metropolitan statistical area (MSA)			<0.001
MSA	25,040,000 (89.0)	247,480,000 (84.7)	
Non-MSA	3,085,000 (11.0)	44,727,000 (15.3)	

Data are n (%), median (interquartile range).

*ED*, emergency department.

### Emergency Department Visit Characteristics

There were no significant differences in presentation of pregnant patients between weekdays and weekends or across seasons. The number of pregnant patients presenting to the ED did not significantly vary across years, even when normalized to total patients in our population. Regional distribution of pregnant patient visits did not differ. Pregnant patients were more likely to present to a hospital in an urban area (89% vs 84.7%, *P* < 0.001). Only 44.8% of patient visits included a pregnancy test, and 44% included an ultrasound; 17.5% of pregnant patient visits included a pelvic exam. Seven percent of pregnant patient visits resulted in hospitalization vs 4.7% of non-pregnant patient visits (*P* < 0.001); and 11.5% of pregnant patient visits included evaluation by a consulting physician.

There was no significant difference in wait time between pregnant vs non-pregnant patient visits. The ED visits generally lasted longer for pregnant patients (33.6% over four hours vs 24.5%, *P* < 0.001). Most (84.4%) patients were seen by an attending physician, without meaningful differences between groups that were seen by other clinicians.

### Multivariable Analysis

Unadjusted and adjusted ORs for associations of patient demographics and hospital characteristics with presentation of pregnant patients compared to non-pregnant patients to the ED are presented in Table 2. In the generated model, age 20–29 years, Hispanic ethnicity, public insurance status, and metropolitan location were significantly associated with visits for pregnant patients. These results held through sensitivity analyses to ensure that imputation in the dataset did not affect our findings.

**Table 2.** Bivariable and multivariable logistic regression models.

	Unadjusted OR	*P*-value	Adjusted OR	*P*-value
Age				
15–19 years	Reference		Reference	
20–29 years	1.61 (1.41–1.83)	<0.001	1.71 (1.48–1.97)	<0.001
30–39 years	0.85 (0.73–0.99)	<0.001	0.91 (0.77–1.07)	<0.001
40–44 years	0.21 (0.16–0.26)	<0.001	0.22 (0.17–0.29)	<0.001
Race/ethnicity				
Non-Hispanic White	Reference		Reference	
Non-Hispanic Black	1.54 (1.35–1.75)	0.07	1.32 (1.15–1.51)	0.74
Hispanic	1.82 (1.60–2.06)	<0.001	1.72 (1.50–1.98)	<0.001
Non-Hispanic Other	1.39 (1.11–1.74)	0.90	1.43 (1.13–1.81)	0.45
Payment source				
Private insurance	Reference		Reference	
Public insurance	1.36 (1.21–1.52)	<0.001	1.24 (1.10–1.39)	<0.001
Self-pay	0.85 (0.73–0.99)	<0.001	0.74 (0.63–0.87)	<0.001
Other	1.05 (0.86–1.29)	0.89	0.90 (0.74–1.08)	0.53
Unknown	1.02 (0.85–1.23)	0.73	0.91 (0.75–1.10)	0.64
Residence				
Private residence	Reference		Reference	
Homeless	0.51 (0.27–0.99)	0.13	0.60 (0.31–1.18)	0.21
Other	0.68 (0.39–1.17)	0.60	0.77 (0.44–1.35)	0.71
Unknown	1.00 (0.71–1.39)	0.12	1.10 (0.74–1.65)	0.17
ED visit day				
Weekend	0.99 (0.90–1.09)	0.86	0.97 (0.88–1.08)	0.60
Weekday	Reference		Reference	
ED visit season				
Fall	Reference		Reference	
Winter	1.14 (0.96–1.36)	0.29	1.14 (0.95–1.38)	0.33
Spring	1.05 (0.88–1.26)	0.72	1.04 (0.87–1.25)	0.52
Summer	1.11 (0.94–1.31)	0.50	1.14 (0.95–1.38)	0.25
Geographic region				
Northeast	Reference		Reference	
Midwest	1.20 (1.01–1.43)	0.21	1.28 (1.09–1.52)	0.02
South	1.16 (0.95–1.42)	0.55	1.26 (1.04–1.52)	0.09
West	1.12 (0.95–1.32)	0.98	1.03 (0.86–1.22)	0.09
Metropolitan statistical area (MSA)				
MSA	1.47 (1.22–1.76)	<0.001	1.41 (1.18–1.67)	<0.001
Non-MSA	Reference		Reference	

*OR*, odds ratio; *ED*, emergency department.

## DISCUSSION

In this study, using data available from NHAMCS, we estimated that there are 2.77 million ED visits for pregnant patients annually in the United States. Most commonly, women presenting to the ED with pregnancy are between the ages of 20–29 years, publicly insured, and identify as Black or Hispanic. Of these pregnant patient visits, we estimate that 13.3% of these resulted in a new diagnosis of pregnancy in the ED, equivalent to approximately 370,000 pregnancies first identified in the ED annually.

We found that 8.6% of visits among women between ages 15–44 years were for pregnant patients, which generally aligns with previously reported values; however, there was a large amount of variability in reported figures.^7^^–^^9^ Benson et al performing a similar evaluation for patients presenting for early pregnancy loss reported approximately 900,000 visits annually, which would represent 32% of the 2.77 million pregnant patient visits we describe.^5^ This is higher than the often reported rate of 20% early pregnancy loss, which we suspect is due to early pregnancy loss being a common reason for ED presentation.^19^

Our data shows that pregnant patients seeking care in the ED are more likely to be Black, Hispanic. or publicly insured. These populations are less likely to receive routine prenatal care and have a higher rates of pregnancy-related morbidity and mortality when compared to White patients.^20^ Furthermore, unintended pregnancy rates are higher and the rate of referral for desired family planning services is lower in these patient groups.^21^^–^^23^ Currently more than one half of abortions are among women of color despite these patients experiencing greater barriers to accessing family planning services.^24^ Patients on Medicaid similarly have challenges accessing abortion care due to limited coverage for these services.^24^ The *Dobbs* decision is likely to exacerbate these disparities and may disproportionately affect pregnant patients presenting to the ED.^24^ Future studies should investigate these impacts as legislation changes and examine how ED presentations and care differ between states that enact restrictive abortion legislation compare to states without restriction.

Our results suggest that a large proportion (13.3%) of pregnant patients who seek care in the ED are first diagnosed during their ED encounter. Based on historical data, half of these pregnancies are unplanned, and half would end in abortion in the pre-*Dobbs* era.^21^ Discovery of these pregnancies in the ED offers an opportunity for counseling and referral to available abortion services if desired. This is especially important in states where strict restrictions on gestational age for legal abortion exist. These patients may face barriers to care and delays in care following ED discharge, suggesting a critical need for counseling and linkage to care during the ED encounter.

Nationwide access to these reproductive healthcare services is supported by the American College of Emergency Physicians. Although the ED is taking a larger role in offering this care, further research is required to identify the needs of this population.^25^^,^^26^ Specifically, future studies could directly measure the rate of new pregnancy diagnosis in the ED, determine counseling practices among emergency clinicians, and examine how these patients are linked to care if a pregnancy is undesired. This data, along with comparisons between states with varying degrees of legislation change, could help inform policy changes.

## LIMITATIONS

Results are based on data from the NHAMCS, which has several, well-reported limitations.^18^ Although the NHAMCS makes great efforts to include a representative sample, it is possible that the included visits are not completely representative of ED visits nationwide. Nevertheless, the NHAMCS is the largest dataset to date with population-based estimates of ED visits in the US. Non-response rate for items in the NHAMCS may also bias results; however, all our variables of interest had non-response rates that fell within acceptable margins, and those with higher non-response rates (race and ethnicity) were evaluated with sensitivity testing to ensure imputed values did not compromise results.

We defined visits with pregnant patients in our population by pregnancy-related ICD-9 and -10 diagnoses, which may have been entered in error for non-pregnant patients. Visits with an incidental pregnancy diagnosis were based on triage data and pregnancy testing, which may have misclassified pregnancies as incidental or failed to identify other incidental pregnancies not captured. To obtain a conservative estimate, we excluded patient presentations for vaginal bleeding, which may have raised clinician or patient suspicion of pregnancy. Due to the nature of the dataset we analyzed, we were not able to provide definitive information about completion of a previous pregnancy test or ultrasound, nor about the patient’s suspicion for pregnancy, which would be preferred markers for identifying new pregnancy diagnoses.

Finally, we were unable to provide information about whether these pregnancies were desired, whether patients had established care with an obstetrician, or the outcomes of these pregnancies.

## CONCLUSION

Our study reveals that pregnant patients make up 3% of ED visits annually. Given recent legislative changes concerning reproductive healthcare, these patients could be significantly impacted. The ED, often seen as the healthcare system’s safety net, provides crucial care that might not be available elsewhere. With the possibility of pregnant patients turning more often to the ED for care, there is an urgent need to develop and implement educational and policy strategies that support these patients in navigating the increasingly complex realm of family planning services.

## Supplementary Information




