# Supplementary material for: Prevalence and Characteristics of Emergency Department Visits by Pregnant People: An Analysis of a National Emergency Department Sample (2010–2020)
Source: West J Emerg Med. 2024 Apr 2;25(3):436–43. doi: 10.5811/westjem.60461 (PMC11112670; doi:10.5811/westjem.60461)
Supplement: Supplementary file 1 [file wjem-25-436-s001.docx]

**Appendix 1:** ICD-9 and ICD-10 codes used for defining pregnancy

**ICD-10 (2016-2020)**

O00-O08: Pregnancy with abortive outcome

O09-O09: Supervision of high risk pregnancy

O10-O16: Edema, proteinuria and hypertensive disorders in pregnancy

O20-O29: Other maternal disorders predominately related to pregnancy

O30-O48: Maternal care related to the fetus and amniotic cavity and possible delivery problems

O94-O9A: Other obstetric complications, not elsewhere classified

Z33: Pregnant state

Z34: Encounter for supervision of normal pregnancy

**ICD-9 (2010-2015)**

630-679: Complications of pregnancy, childbirth, and the puerperium

V22: Normal pregnancy

V23: Supervision of high risk pregnancy
